# The Value of Texture Analysis of Multi-parameter MRI Images in Distinguishing Benign and Malignant Lesions of O-RADS MRI 4 Lesions

**DOI:** 10.7150/ijms.107452

**Published:** 2025-02-26

**Authors:** Yan Lei, Hanzhou Tang, Lianlian Liu, Tingting Zheng, Yuan Zhang, Tong Chen, Junkang Shen, Bin Song

**Affiliations:** 1 Department of Radiology, Minhang Hospital, Fudan University, 170 Xinsong Road, Shanghai, 201199, Shanghai, People's Republic of China.; 2 Department of Radiology, The Second Affiliated Hospital of Soochow University, Soochow University, 1055 Sanxiang Road, Suzhou, 215004, Jiangsu, People's Republic of China.

**Keywords:** Magnetic resonance imaging, Texture analysis, Ovarian lesion, Ovarian-adnexal reporting and data system MRI (O-RADS MRI), Benign and malignant lesions

## Abstract

**Objectives:** To investigate the diagnostic performance of texture analysis using multi-parameter MRI in distinguishing between benign and malignant lesions with ovarian-adnexal magnetic resonance imaging report and data system (O-RADS MRI) score 4.

**Methods:** A retrospective analysis was conducted of 57 lesions with an O-RADS MRI score of 4, of which 26 were benign and 31 were malignant. Based on the T2WI, ADC, and CE_T1WI, the textural features of the entire lesion were extracted. The minimum redundancy maximum relevance (mRMR) method was used to select features, and the random forest (RF) algorithm was used to construct four prediction models: T2WI, ADC, CE_T1WI, and the combined models. Ten-fold cross-validation was used to verify the model prediction performance, and receiver operating characteristic (ROC) analysis was used to evaluate the model performance, including area under the curve (AUC), accuracy, sensitivity, specificity, positive predictive value (PPV), and negative predictive value (NPV).

**Results:** 3474 texture features were extracted from the ADC, T2WI, and CE_T1WI images. ADC, T2WI, CE_T1WI, and combined models were constructed. Each model contained ten texture features. The AUC of the ADC, T2WI, CE_T1WI, and combined models were 0.749 (95% CI: 0.621-0.876), 0.671 (95% CI: 0.524-0.818), 0.786 (95% CI: 0.662-0.909), and 0.860 (95% CI: 0.76-0.959), respectively. The AUC of the combined model was significantly higher than those of the other three groups. The accuracy, sensitivity, specificity, PPV, and NPV of the combined model in distinguishing benign and malignant lesions with an O-RADS MRI score of 4 were 75.9%, 77.8%, 74.1%, 72.4%, and 79.3%, respectively.

**Conclusion:** Texture analysis of multi-parameter MRI can improve the diagnostic efficiency of distinguishing benign and malignant lesions with an O-RADS MRI score of 4 and provide some help in clinical decision-making.

## Introduction

Ovarian tumors are the most common tumors of the female reproductive system and are not easily detected, especially in the early stages. Owing to their high morbidity and mortality, distinguishing between benign and malignant ovarian tumors is of great importance^1^. Currently, imaging remains the primary method of ovarian tumor detection. Ultrasound has the advantages of painlessness, simplicity of operation, and high repeatability in diagnosing ovarian tumors and is widely used. However, approximately 18%-31% of ovarian tumors cannot be accurately diagnosed using ultrasound examination^2^, ^3^.

Compared with ultrasound, magnetic resonance imaging (MRI) shows better anatomical resolution and soft-tissue contrast imaging^4^ and is also a multi-parameter imaging technique. In this context, the ADNEX-MR score was introduced in 2013 as a risk assessment and adnexal lesion scoring system^4^, and its name was changed to ovarian-adnexal reporting and data system MRI (O-RADS MRI) score in 2020^5^. The O-RADS allows for the stratification of the malignancy risk of adnexal masses based on the lesion's composition, the signal intensity characteristics, and the solid tissue's enhancement pattern. The O-RADS MRI risk scoring system has promising diagnostic efficacy and reproducibility, but 10.8% to 12.5% of ovarian adnexal masses malignancies cannot be determined. In patient management dilemmas, O-RADS MRI 1-3 (normal to low-risk) and O-RADS MRI 5 (high-risk) usually do not lead to difficulties. Considering the wide range of malignant tumors in such lesions, the adnexal lesions with O-RADS MRI 4 (moderate risk) have been considered as the system's Achilles' heel. The positive predictive value (PPV) of malignant lesions with an O-RADS MRI risk score of 4 was only 50%^6^. In addition, when nondynamic multi-phase contrast-enhanced (CE) MRI is used to evaluate adnexal lesions, the PPV of O-RADS MRI 4 is still unclear and requires further study^4^, ^5^, ^7^. A lower PPV may lead to a considerable number of patients with benign adnexal tumors undergoing potentially unnecessary or excessive surgical intervention. Consequently, more criteria are required to sub-stratify O-RADS MRI 4 for better risk stratification. The O-RADS MRI risk-scoring system is still in the early stages of use, and further research is necessary to optimize and develop it. The O-RADS MRI score is principally based on MRI's morphological and functional manifestations and assesses tumors through macroscopic observation of lesion morphological characteristics and semi-quantitative evaluation. It has limited subjectivity and lacks objective quantitative data analysis.

Texture analysis (TA) is a mathematical method that systematically and quantitatively analyzes image features to evaluate tissue gray-scale patterns, locations, and the relationship between pixels and voxels. It can quantify tumor heterogeneity and microstructure that the human visual system cannot detect^8^, ^9^. Recently, texture analysis has been combined with various imaging methods to predict the pathological classification, staging, and postoperative prognosis of gynecological tumors^10^, ^11^. Wang *et al.*^12^ used a multimodal MRI texture analysis model to predict the prognosis of ovarian cancer and found that a model based on T2WI had the best performance. Ye *et al.*^13^ explored the value of MRI-based whole-tumor texture analysis in distinguishing borderline and malignant epithelial ovarian tumors and found that the area under the receiver operating characteristic curve (AUC) of the combined model of texture features and clinical data was 0.962. There have been few reports on using TA to improve the O-RADS MRI scores to 4. Therefore, this study aimed to explore the value of TA based on multimodal MRI images to improve the O-RADS MRI score to 4.

## Materials and methods

### Patients and study setting

This retrospective study was conducted between March 2015 and December 2022 at the Radiology Department of Minhang Hospital, affiliated with Fudan University, China. It included 147 patients with 155 adnexal masses confirmed surgically and pathologically. The Institutional Review Board of Minhang Hospital, affiliated with the Fudan University Review Board, approved this retrospective study, and informed consent was not required.

The inclusion criteria were pelvic MRI enhancement examination within 4 weeks before surgery and a final O-RADS MRI lesion score of 4 points. The exclusion criteria were poor image quality, inability to be used for evaluation and analysis, missing or incomplete MRI images, incomplete clinical and pathological data, and preoperative radiation or chemotherapy. The final cohort comprised 52 patients with 57 adnexal masses.

### Magnetic resonance imaging

All MRI examinations were performed on a 3.0-T system (uMR780 3.0 T MRI; United Imaging Healthcare, Shanghai, China) and a 1.5-T system (EXCITE HD 1.5T MRI; GE Healthcare, Milwaukee, WI, USA) using a phased-array coil. Fasting was done for 4-6 hours before examination to limit artifacts caused by intestinal peristalsis. The scan range of the pelvis was from the iliac crest to the pubic symphysis and was adjusted to cover the entire size of the adnexal lesion. The contrast agent Magnevist was given at a dose of 0.2 mL per kilogram of body weight using a power injector at a rate of 2 mL/s, followed by 15 mL of normal saline to flush the tubing. Our institutional standard MRI protocol included the following sequences: axial and T1 fast spin-echo (FSE) weighted imaging (WI) with and without fat saturation; sagittal and axial T2 FSE WI; axial diffusion-weighted images (DWI) with b-values of 0, 800, 1000 s/mm^2^ to obtain apparent diffusion coefficient (ADC) maps; dynamic T1weighted 3D gradient-echo with fat saturation in the axial and sagittal plane during contrast uptake and delayed post-contrast T1-weighted 3D gradient echo with fat saturation in the axial plane. The scanning sequences are presented in Table 1.

### Image processing

Two radiologists with 5 and 15 years of experience in female pelvic imaging analyzed all images independently. Both radiologists were blinded to the clinical and histological data, and retrospectively classified the adnexal masses according to the O-RADS MRI scoring system published by Thomassin *et al.* in January 2020^5^. If there is a disagreement between the two readers a consensus can be reached through discussion.

According to previously published studies, the following MRI characteristics were analyzed for each adnexal mass^14^-^17^: distribution location (unilateral, bilateral), shape (regular, irregular), boundary (clear, unclear), lesion diameter (maximum cross-sectional diameter of the lesion), lesion composition (cystic, cystic solid, solid), T1WI image signal strength, T2WI lipid-pressure image signal intensity, DWI signal strength, enhancement mode, ascites (with or without), lymph nodes (with or without). The signal intensity of the endometrium was used as the reference standard for the signal intensity of lesion DWI, and the degree of enhancement of the myometrium was used as the reference standard for the degree of enhancement of the lesion. Patients with a score of 4 on the O-RADS non-dynamic enhanced MRI were enrolled.

Adnexal lesion segmentation was performed using ITK-SNAP software (http://www.itk-snap.org). Regions of interest (ROI) were manually segmented independently by two radiologists with 5 and 15 years of experience in MRI image evaluation, respectively, layer by layer for the entire lesion. Both radiologists reached a consensus in cases with obscured tumor margins by performing additional image analyses.

### Feature extraction and selection

An Artificial Intelligence Kit (A. K., GE Healthcare) was used to analyze the volume of interest (VOI) and extract lesion texture features. A total of 3474 features were obtained, including shape features, first-order statistics, gray-level co-occurrence matrix (GLRLM, gray-level run length matrix (GLSZM, gray-level size zone matrix (GLDM, gray-level dependence matrix (GLDM), and Laplace of Gaussian transformed features (LoG). PyRadiomics (http://www.radiomics.io/pyradiomics.html) was used to extract 704 wavelet transform features. Detailed information on the textural features is presented in Table 2.

### Feature selection and model construction

The intraclass correlation coefficient (ICC) of the textural features of 30 cases was randomly selected to evaluate the intra- and inter-observer repeatability of lesion segmentation. Radiologist 1 manually plotted the VOI twice within two weeks for intra-observer ICC assessment. Radiologist 2 also outlined the VOI, and the extracted texture features were used to evaluate the ICC between observers. ICC > 0.75 showed good consistency.

The Mann-Whitney U test was used for univariate analysis. The texture features were retained when the intra-and inter-observer ICCs were greater than 0.75 and *p* < 0.1. Subsequently, the mRMR method was used to select the feature subset, and features with minimum redundancy and maximum correlation were retained. Subsequently, four prediction models of T2WI, ADC, T1WI, and three combined models were constructed using the random forest (RF) algorithm. Ten-fold cross-validation was used to verify the model's predictive performance, and ROC curve analysis was used to evaluate the model's performance. The areas under the curve AUC, accuracy, sensitivity, specificity, PPV, and NPV were also recorded.

### Judgment of pathological results

After surgical resection, all specimens were formalin-fixed, dehydrated, paraffin-embedded, sectioned, and stained with hematoxylin-eosin staining (HE staining). Two senior pathologists observed the sections independently. If HE staining cannot be used for diagnosis, immunohistochemistry can be used to further determine the pathological type.

### Statistical analysis

All statistical analyses were conducted using SPSS statistical software (version 26.0; IBM Corp., Armonk, NY, USA) and R software (version 4.2.0; http://www.r-project.org). The Kolmogorov-Smirnov test determined whether the measurement data conformed to a normal distribution. The measurement data obeying the normal distribution was tested by independent sample T-test, expressed as mean ± standard deviation (x ± SD). Measurement data that did not follow a normal distribution were tested using the rank sum test and expressed as median and quartile spacing [M (P25, P75)]. The chi-square or Fisher's exact test was used to compare intergroup differences in categorical variables. The intragroup correlation coefficient was used for the observer consistency test. ICC assessed the consistency between observers using the following criteria: excellent 0.75-1.00; good 0.50-0.75; acceptable 0.25-0.50; very poor 0-0.25. *p* < 0.05 was considered significant.

## Results

### Clinical baseline data

The patients' baseline clinical and pathological data are shown in Tables 3 and 4. This study enrolled 52 patients with 57 lesions (five cases of bilateral lesions) aged 17-86 years, with an average age of 51.5 years. Pathological results: 26 cases (45.6%) were benign lesions (8 cases of serous cystadenoma, 7 cases of mucinous cystadenoma, 3 cases of goiter, 3 cases of ovarian fibroma, 1 case of Brenner tumor, 2 cases of theca cell tumor, 2 cases of ovarian salpingitis mass), 31 cases (54.4 %) were malignant (3 cases of borderline serous tumor, 5 cases of borderline mucinous tumor, 4 cases of serous adenocarcinoma, 1 case of mucinous adenocarcinoma, 3 cases of clear cell carcinoma, 5 cases of endometrioid carcinoma, 2 cases of granular cell tumor, 1 case of malignant teratoma, and 7 cases of metastatic carcinoma). Among the 57 patients, 26 were postmenopausal, 31 were premenopausal, 15 were postmenopausal in patients with benign tumors, and 11 were postmenopausal in patients with malignant tumors. Among the 57 patients, 34 had elevated serum carbohydrate antigen 125(CA125) levels, one had elevated alpha-fetoprotein (AFP) levels, and five had elevated serum carcinoembryonic antigen (CEA) levels. There were no significant differences in age, serum (CA125) level, alpha-fetoprotein (AFP) level, carcinoembryonic antigen (CEA) level, and menopause between patients with benign and malignant tumors (*p* > 0.05).

### Texture feature selection and model construction

A total of 3474 texture features were extracted from ADC, T2WI, and CE_T1WI images. Significant texture features were selected by single-factor analysis, and the ADC, T2WI, T1WI enhanced, and combined models were established using the mRMR and RF algorithms. Each model contained ten texture features. The ten texture features in the ADC model include two first-order features, two LoG features, and six wavelet features. The ten texture features in the T2WI model included three LoG features and seven wavelet features. Ten texture features in the T1WI enhancement model included five first-order features, one LoG feature, and four wavelet features. Ten texture features in the combined model included three first-order features, one LoG feature, and six wavelet features. The ICC of the texture features of the model was greater than 0.75, and the results of the single-factor analysis of the selected features of the four models are shown in Table 5.

**Figure 1:**
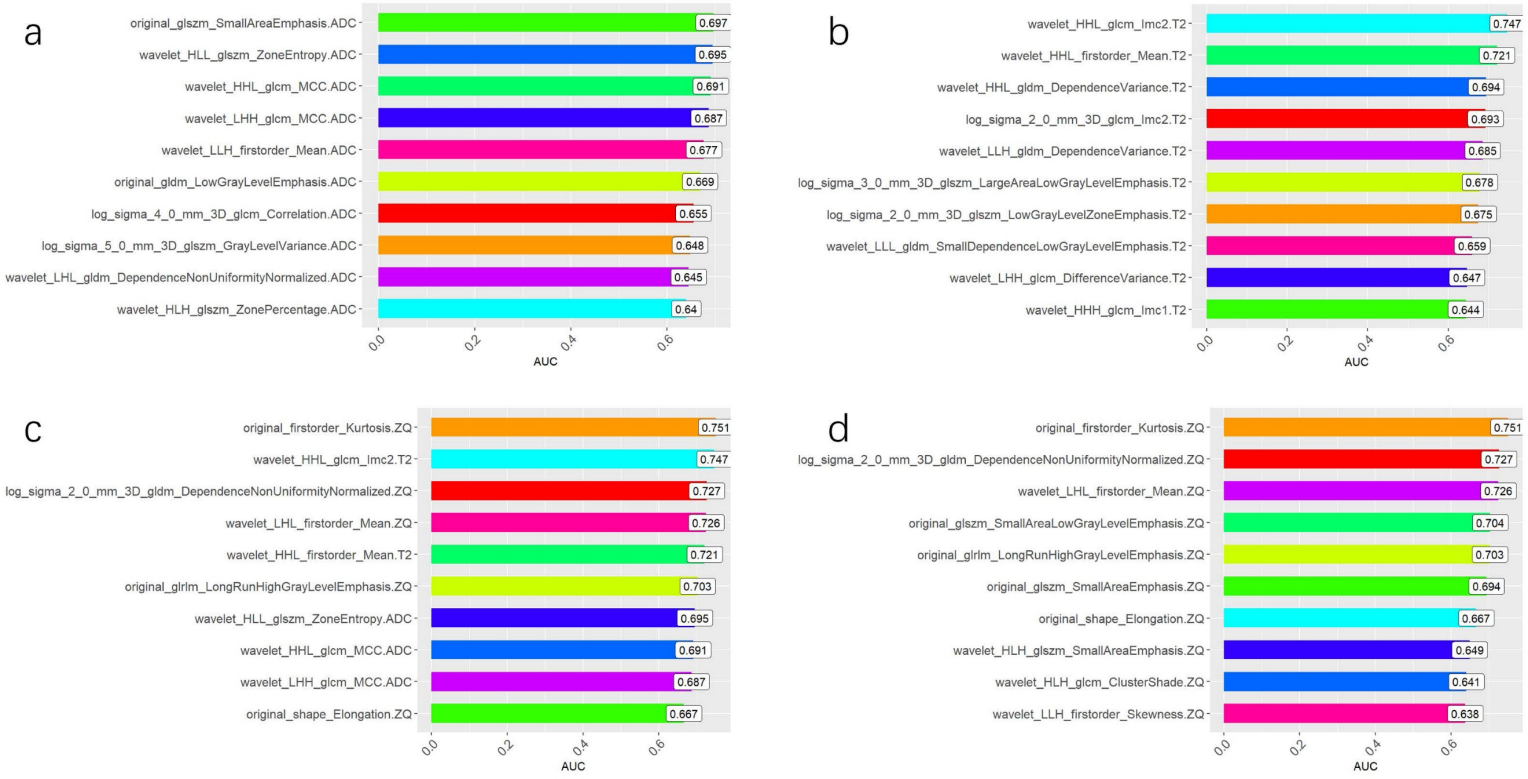
** Independent predictive factors AUC of four models. (A)** Comparison of AUC of 10 independent predictive factors of ADC model. **(B)** Comparison of AUC of 10 independent predictive factors of T2 WI model. **(C)** Comparison of AUC of 10 independent predictive factors of the combined model. **(D)** Comparison of AUC of 10 independent predictive factors of CE_T1WI model.

**Figure 2:**
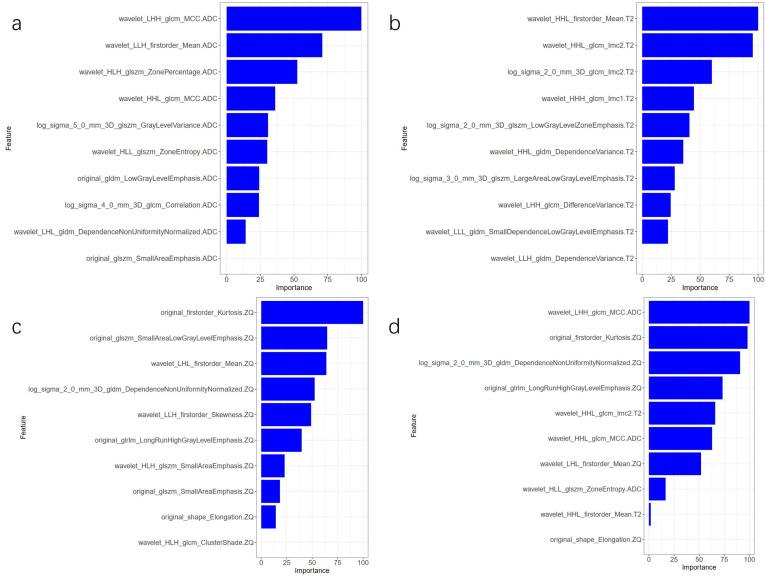
** Comparison of the importance of four model parameters in the model. (A)** ADC model; **(B)** T2WI model; **(C)** CE_T1WI model; **(D)** combined model.

**Figure 3:**
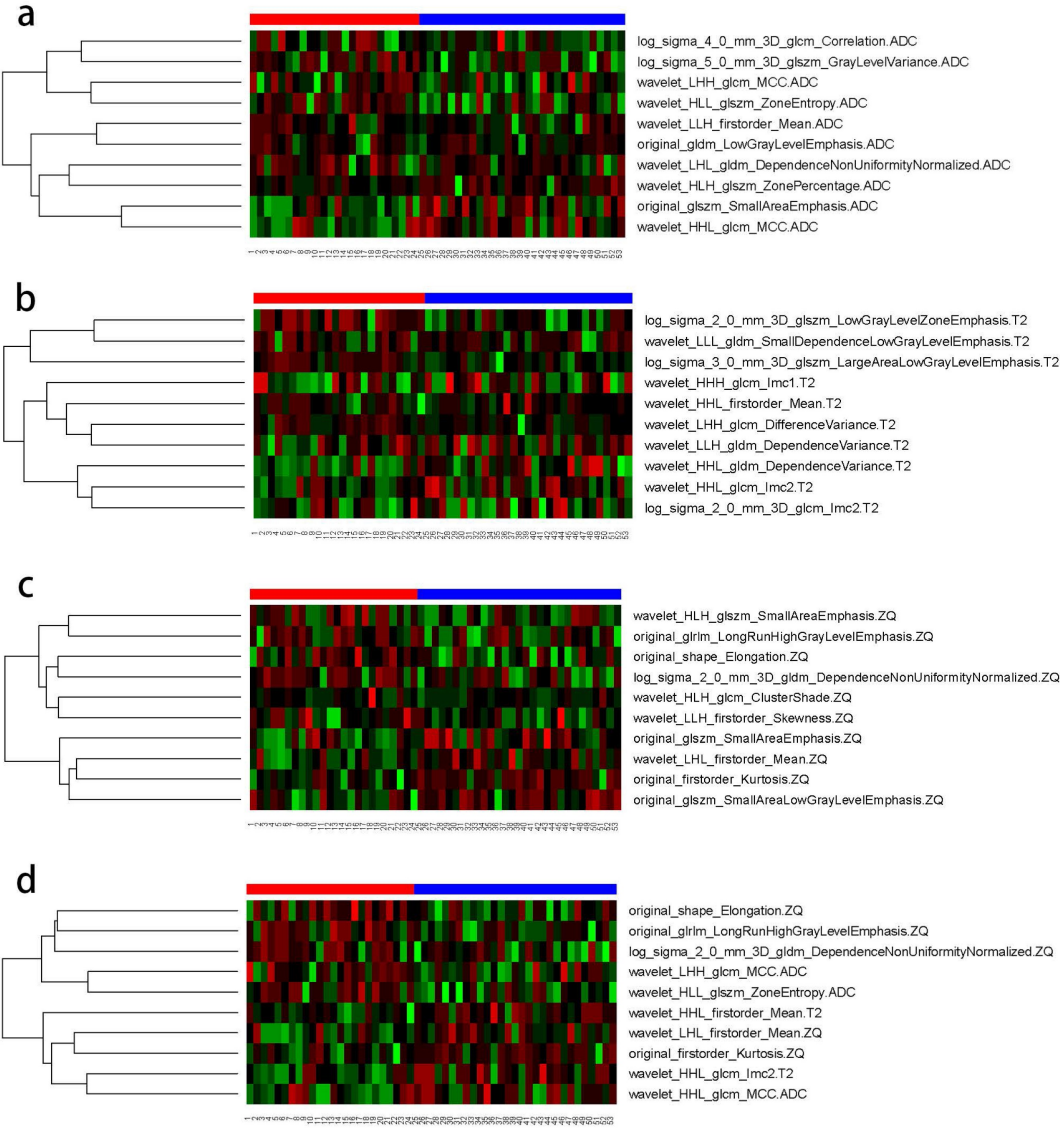
** Correlation between the expression of 10 independent predictive factors in four models' heat maps and tumor malignancy. (A)** ADC model; **(B)** T2WI model; **(C)** CE_T1WI model; d. combined model.

The AUC comparison of the 10 independent predictive factors selected by the four models is shown in Figure 1, and the important components of the 10 independent predictive factors in the four models are shown in Figure 2, among which the wavelet_LHH_glcm_MCC.ADC features account for the largest proportion of the selected ten features in the combined model. The correlation between the expression of the 10 independent predictive factors in the heat map of the four models and malignancy is shown in Figure 3.

### Prediction performance of the model

**Figure 4:**
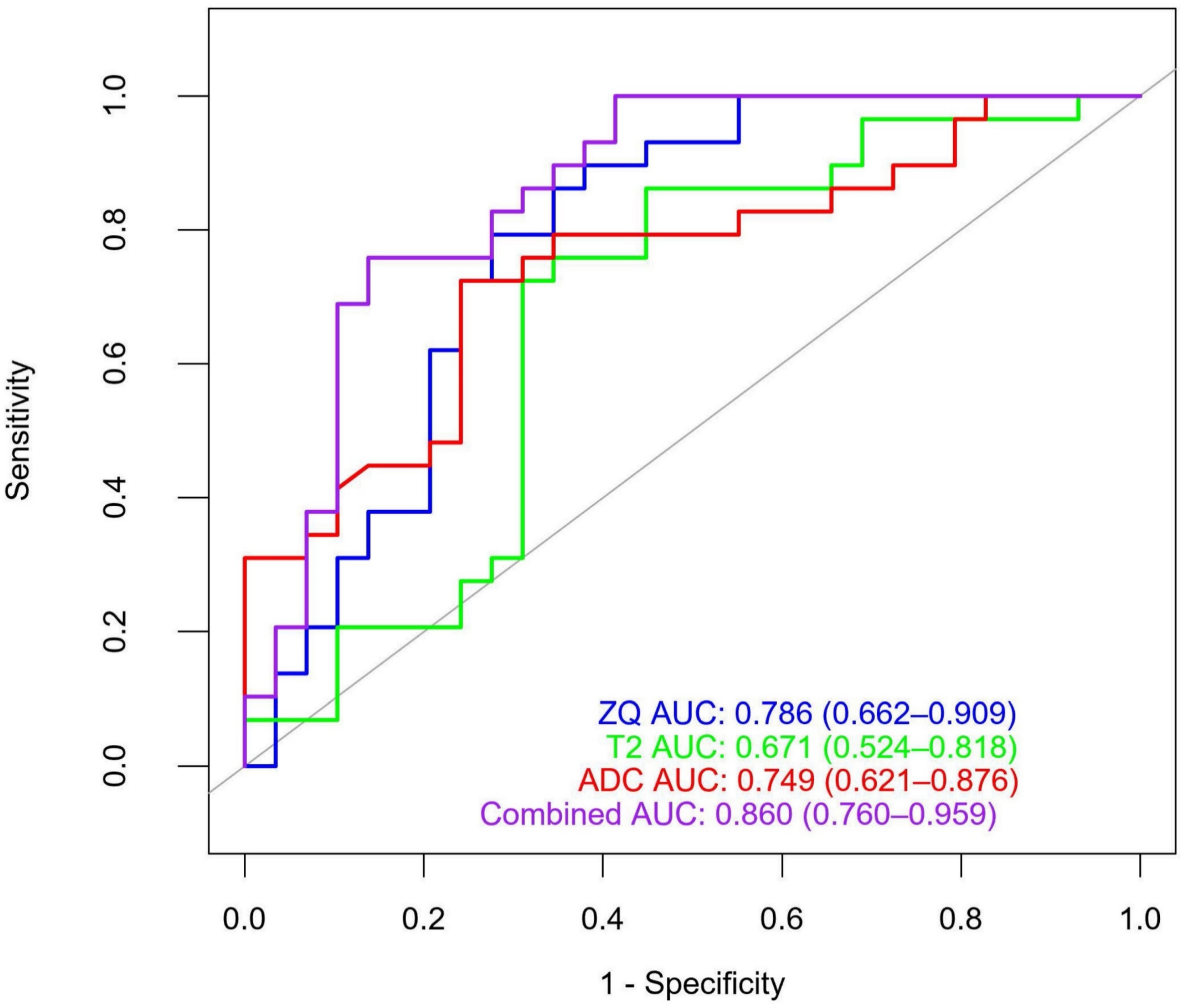
ROC curves of four different models.

**Figure 5:**
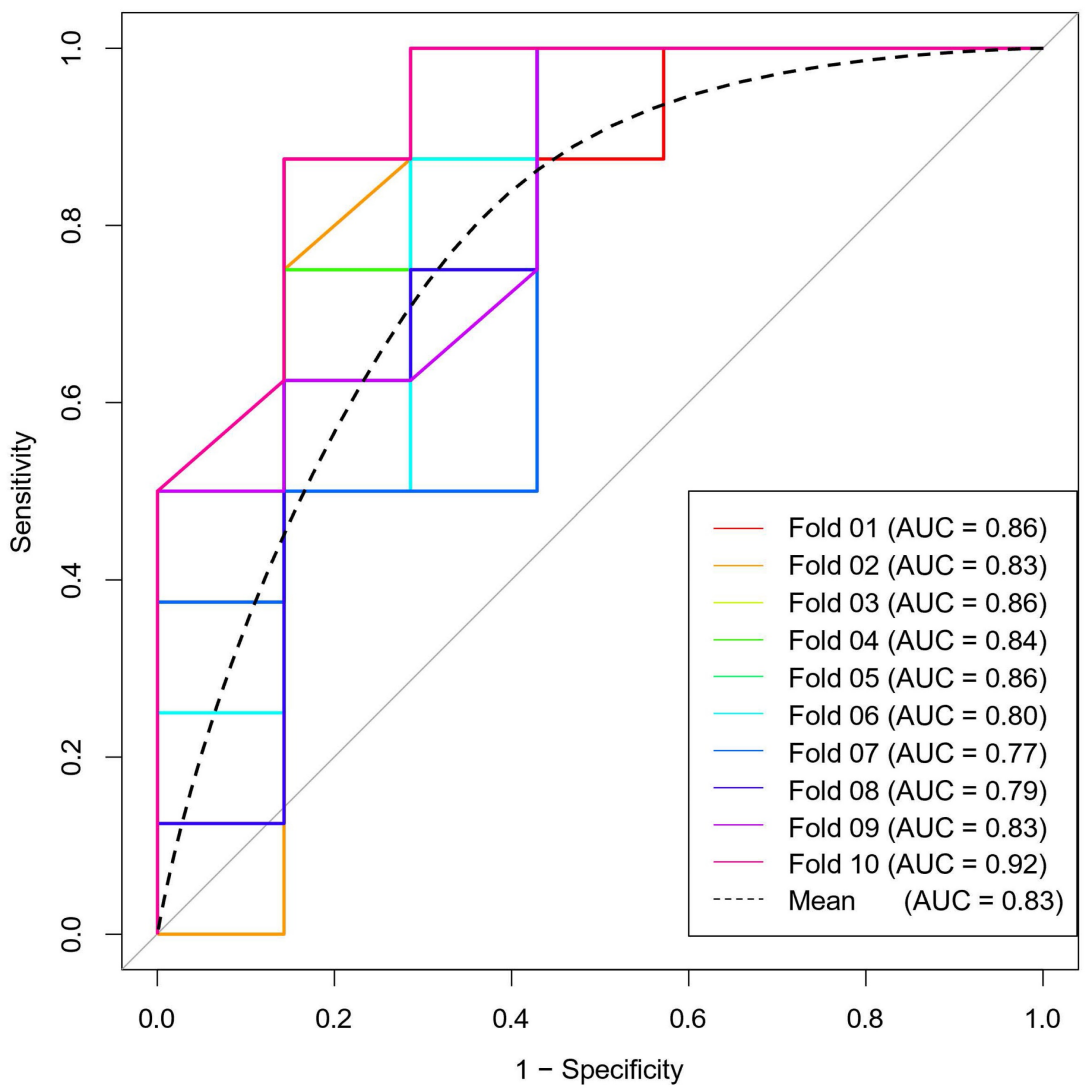
ten-fold cross-validation diagram.

The ROC curves for the four models are shown in Figure 4. The AUC of the ADC, T2WI, and CE_T1WI models were 0.749 (95 % CI: 0.621-0.876), 0.671 (95 % CI: 0.524-0.818), and 0.786 (95 % CI: 0.662-0.909), respectively. The Delong test showed that there was no difference in AUC among the three model groups (T2WI vs CE_T1WI model, *p* = 0.101; T2WI vs. ADC model, *p* = 0.347; CE_T1WI vs ADC model, *p* = 0.597). The AUC of the combined model was 0.860 (95% CI: 0.76-0.959). Delong tests showed that the AUC between the T2WI, ADC, and combined models was statistically significant (T2WI and combined model, *p* = 0.004; ADC vs. combined model, *p* = 0.038; CE_T1WI model vs combined model *p* = 0.070). The AUC of the combined model was significantly higher than those of the other three groups. A ten-fold cross-validation of the combined model is shown in Figure 5. The combined model exhibited the best diagnostic performance, with an accuracy of 0.759. After ten-fold cross-validation, it performed well, with an accuracy of 0.713. Table 6 presents the diagnostic performance of the four models. The combined model had the best performance, with accuracy, sensitivity, specificity, PPV, and NPV of 75.9%, 77.8%, 74.1%, 72.4%, and 79.3%, respectively.

## Discussion

This study evaluated the diagnostic performance of TA in improving the diagnostic performance of O-RADS 4. Our results showed that the texture features from the T2WI, ADC, and CE_T1WI images significantly differed between benign and malignant O-RADS MRI 4. The combined model based on textural features showed promising efficiency in distinguishing between benign and malignant O-RADS 4 lesions (AUC = 0.86).

The O-RADS MRI risk scoring system is based on the MRI scoring system for adnexal lesions (ADNEX MR scoring system) developed by Thomassin *et al.* in 2013^4^. It was developed and published by a multidisciplinary, international expert committee under the guidance of the American College of Radiology in 2020 as the most comprehensive standard for MRI evaluation of ovarian and adnexal lesions. A meta-analysis by Qing Zhang *et al.*^18^ found that O-RADS US and O-RADS MRI have high sensitivity for ovarian or adnexal malignancies. However, O-RADS MRI provides higher specificity (90%). A meta-analysis involving 4,012 ovarian adnexal lesions by Stefania Rizzo *et al.*^19^ found that the total malignant probability of O-RADS MRI 4 lesions was 60% (95% CI, 52%-67%). This study quantified the texture features of whole lesions of ovarian tumors based on multi-parameter MRI TA from magnetic resonance ADC maps, T2WI, and CE_T1WI. Our results indicate that the combined model based on the ADC map, T2WI, and T1WI enhanced sequences can improve the diagnostic efficacy between benign and malignant lesions with an O-RADS MRI score of 4. Quantified texture features compensate for empirical diagnoses based on conventional MRI morphology deficiencies. The MRI-based texture feature model provides a new method for noninvasively improving the diagnostic efficacy of O-RADS 4 before surgery, which helps evaluate preoperative treatment strategies.

TA is a new imaging technology that can extract large amounts of data from biomedical images and be studied using TA tools. TA of the MRI images was performed by analyzing the gray tone changes between the image voxels. This method captures spatial and intensity information to identify pathological changes and microstructural heterogeneity within tumors. Because of the heterogeneity of ovarian tumors, texture features based on the entire tumor contain more spatial information and higher sensitivity and specificity than traditional methods based on pre-selected regions of interest. This study used 3D whole-lesion ROI delineation to avoid the limitations of selecting individual layers corresponding to partial lesion areas and maximizing their diversity^20^. 3D TA is a method that utilizes all data dimensions and contains rich information about the internal structure of related objects. Increasing the number of points required for feature calculation to provide a more complete description of the lesion can improve the accuracy of lesion heterogeneity characterization and reduce sampling errors^21^, ^22^.

There were subtle grey differences between the tumor and its surrounding tissues, but the difference was not obvious; areas with more low-frequency and high-frequency information appeared randomly. The wavelet transform relies mainly on multi-resolution analysis to decompose an image into components of different frequencies, followed by data correction. The multi-resolution analysis characteristics of the wavelet transform make it widely applicable to the texture features of non-stationary signals, such as medical images^23^. In this study, among the ten features selected by the combined model, there were six high-order wavelet transform features. TA was performed in the spatial and frequency domains to provide changes in spatial resolution and represent texture on the most appropriate scale. Wavelet transform features enhance the details of an image, which is of great significance in this study. Among the 10 features the combined model selected, three come from GLCM, where the feature wavelet_LHH_glcm_MCC.ADC_DWI_b = 800 accounts for the largest proportion among the selected ten features. The relationship between two pixels can influence the GLCM and calculate the number of occurrences of all possible combined gray values in a specific direction and the distance between them. The GLCM is calculated for multiple directions and distances, retains only the required solutions, and presents the best features. It is one of the most important and in-depth TA methods and many applications. It remains the benchmark method for most TA research^24^-^26^. Fathi Kazerooni *et al.*^27^ showed that GLCM texture parameters based on magnetic resonance ADC maps have value in the differential diagnosis between benign and malignant solid ovarian tumors. Combined with our findings, we assume that GLCM features may provide unique information regarding ovarian tumors.

Since the introduction of radiomics in 2012, TA has been widely used to study ovarian tumors. RA *et al.*^28^ established a prediction model based on the radiomic TA of MRI images of liquid components in ovarian cysts. The prediction model's sensitivity, specificity, and AUC for identifying malignant lesions were 84.62%, 80%, and 0.841, respectively. Zhang *et al.*^29^ established a radiomics model based on the texture features of multiple sequences of MR images (T1WI, T2WI, T2WI_fs, DWI, and T1WI multi-phase enhancement), which had higher diagnostic accuracy than radiologists in differentiating benign and malignant ovarian tumors (90.6% and 83.5%, respectively). The model could distinguish type I and type II epithelial ovarian cancers with an accuracy of 83% and an AUC of 0.85, and its diagnostic performance was better than when the features were applied separately.

A few researchers have attempted to combine MRI radiomics to improve the effectiveness of O-RADS MRI. Hottat *et al.*^30^ applied DWI quantitative analysis, including ROI-ADC and whole lesion-ADC histogram measurements combined with O-RADS MRI 4, and the overall performance of O-RADS was improved. However, no studies have focused on applying MRI-based textural features to improve the performance of O-RADS MRI. Multi-parameter TA based on MRI has been widely used to improve the magnetic resonance stratification management score of breast, prostate, and other tumors^31^-^33^. This study focused on the multi-parameter MRI texture features of magnetic resonance ADC maps, T2WI, and CE_T1WI to improve the effectiveness of O-RADS MRI, which showed good clinical practicability.

Although the TA model based on multi-parameter MRI had good accuracy, this study had some limitations. First, this was a retrospective study, and selection bias may exist. Second, the sample size was small, therefore, a multi-center study with an expanded sample size is necessary to verify the applicability of the results. In addition, although this study explored the preliminary results of MRI image texture analysis to improve the diagnostic efficacy of O-RADS MRI with a score of 4 for benign and malignant tumors, further studies are necessary to confirm the pathological basis.

In summary, the model based on the whole lesion of the ovarian tumor texture of multi-parameter MRI helps improve the diagnostic efficiency of the O-RADS MRI score of 4 for benign and malignant tumors. This approach offers a promising solution to the existing challenges in differentiating O-RADS MRI 4 lesions, while also addressing the variability in clinical expertise among imaging diagnostic physicians—particularly junior practitioners who predominantly rely on conventional MP-MRI morphological features for evaluation. Moving forward, we aim to undertake large-scale, multicenter prospective cohort studies to further assess the practical utility of O-RADS MRI scores in real-world clinical settings. These efforts aim to provide an objective and accurate diagnosis and treatment basis for clinical practice, assists in developing personalized diagnosis and treatment plans, and improves patient prognosis and survival rates.

## Figures and Tables

**Table 1:** MR scanning parameters in detail.

	T1WI	T2WI	T2WI	DWI	3D LAVA/ Dyn 3D T1 fs	3D LAVA/ Dyn 3D T1 fs
Scanning Planes	Axial	Sagittal	Axial	Axial	Axial	Sagittal
Slice thickness(mm)	4	5	5	5	6	6
Intersection gap(mm)	0.8	1-1.5	1-1.5	1	-	-
FOV (mm)	240×220	240×240	240×240	240×240	380×280	380×280
acquisition matrix	320×256	256×256	256×256	128×128	368×312.8	368×312.8
B values (s/mm2)				0-800-1000		
acquisition time	0.06875	0.093055556	0.088194444	0.175694444	0.057638889	0.081944444

**Table 2:** Summary of 1158 Texture Features

feature classification	name	number
Original	Shape	14
	Firstorder	18
	Glcm	24
	Glrlm	16
	Glszm	16
	Gldm	14
LOG	Firstorder	72
	Glcm	96
	Glrlm	64
	Glszm	64
	Gldm	56
wavelet	LLH	88
	LHL	88
	LHH	88
	HLL	88
	HLH	88
	HHL	88
	HHH	88
	LLL	88

Abbreviation: Glcm, gray level co-occurrence matrix; Glrlm, gray level run length matrix; Glszm, gray scale region matrix; Gldm, gray level dependence matrix; Log, Laplace of Gaussian transformed features.

**Table 3:** Clinical baseline data

feature	benign	malignant	*p* value
	(n=26)	(n=31)	
Age, average ± SD, year	54.85 ± 17.55	49.35 ± 12.22	0.171
CA125			0.054
Up-regulated	26 (55.2)	10 (45.5)	
Normal	32 (44.8)	12 (54.5)	
CEA			0.094
Up-regulated	0 (0.0)	5 (16.1)	
Normal	26 (100.0)	25 (83.9)	
AFP			1.000
Up-regulated	0 (0.0)	1 (3.2)	
Normal	26 (100.0)	30 (96.8)	
menopause			0.097
postmenopausal	15 (57.7)	11 (35.5)	
premenopausal	11 (42.3)	20 (64.5)	

The data were expressed as a percentage of the number of patients in parentheses; SD, standard deviation

**Table 4:** Statistical table of pathological data

Malignant degree	Pathological type	Counts (%)
Benign	serous cystadenoma	8 (14)
	mucinous cystadenoma	7 (12.3)
	goiter	3 (5.3)
	ovarian fibroma	3 (5.3)
	Brenner tumor	1 (1.7)
	theca cell tumor	2 (3.5)
	ovarian salpingitis mass	2 (3.5)
	total	26 (45.6)
Malignant	borderline serous tumor	3 (5.3)
	borderline mucinous tumor	5 (8.7)
	serous adenocarcinoma	4 (7.0)
	mucinous adenocarcinoma	1 (1.7)
	clear cell carcinoma	3 (5.3)
	endometrioid carcinoma	5 (8.7)
	granular cell tumor	2 (3.5)
	malignant teratoma	1 (1.7)
	metastatic carcinoma	7 (12.3)
	total	31 (54.4)

The data are expressed as a percentage in parentheses.

**Table 5:** Single factor analysis results of selected variables for ADC, T2WI, CE-T1WI, and combined model

model	variable	*p* value	AUC (95% confidence interval)				
ADC model	original_glszm_SmallAreaEmphasis.ADC	0.014*	0.697 (0.553-0.840)				
	wavelet_HLL_glszm_ZoneEntropy.ADC	0.015*	0.695 (0.548-0.842)				
	wavelet_HHL_glcm_MCC.ADC	0.017*	0.691 (0.536-0.845)				
	wavelet_LHH_glcm_MCC.ADC	0.020*	0.687 (0.530-0.843)				
	wavelet_LLH_firstorder_Mean.ADC	0.028*	0.677 (0.520-0.833)				
	original_gldm_LowGrayLevelEmphasis.ADC	0.036*	0.669 (0.514-0.824)				
	log_sigma_4_0_mm_3D_glcm_Correlation.ADC	0.050*	0.655 (0.499-0.811)				
	log_sigma_5_0_mm_3D_glszm_GrayLevelVariance.ADC	0.065	0.648 (0.495-0.800)				
	wavelet_LHL_gldm_DependenceNonUniformityNormalized.ADC	0.071	0.645 (0.486-0.803)				
	wavelet_HLH_glszm_ZonePercentage.ADC	0.081	0.640 (0.489-0.791)				
T2WI model	wavelet_HHL_firstorder_Mean.T2	0.005*	0.721 (0.579-0.863)				
	wavelet_HHL_glcm_Imc2.T2	0.002*	0.747 (0.601-0.884)				
	wavelet_HHL_gldm_DependenceVariance.T2	0.015*	0.693 (0.542-0.846)				
	log_sigma_2_0_mm_3D_glcm_Imc2.T2	0.017*	0.692 (0.545-0.839)				
	wavelet_LLH_gldm_DependenceVariance.T2	0.021*	0.685 (0.541-0.829)				
	log_sigma_3_0_mm_3D_glszm_LargeAreaLowGrayLevelEmphasis.T2	0.026*	0.678 (0.530-0.826)				
	log_sigma_2_0_mm_3D_glszm_LowGrayLevelZoneEmphasis.T2	0.020*	0.675 (0.527-0.824)				
	wavelet_LLL_gldm_SmallDependenceLowGrayLevelEmphasis.T2	0.047*	0.659 (0.509-0.809)				
	wavelet_LHH_glcm_DifferenceVariance.T2	0.068	0.646 (0.496-0.796)				
	wavelet_HHH_glcm_Imc1.T2	0.073	0.643 (0.487-0.800)				
CE-T1WI model	original_firstorder_Kurtosis.ZQ	0.001*	0.751 (0.613-0.889)				
	log_sigma_2_0_mm_3D_gldm_DependenceNonUniformityNormalized.ZQ	0.004*	0.727 (0.584-0.869)				
	wavelet_LHL_firstorder_Mean.ZQ	0.005*	0.725 (0.583-0.868)				
	original_glszm_SmallAreaLowGrayLevelEmphasis.ZQ	0.011*	0.704 (0.549-0.858)				
	original_glrlm_LongRunHighGrayLevelEmphasis.ZQ	0.014*	0.702 (0.556-0.849)				
	original_glszm_SmallAreaEmphasis.ZQ	0.015*	0.694 (0.547-0.840)				
	original_shape_Elongation.ZQ	0.038*	0.667 (0.519-0.814)				
	wavelet_HLH_glszm_SmallAreaEmphasis.ZQ	0.063	0.649 (0.498-0.800)				
	wavelet_HLH_glcm_ClusterShade.ZQ	0.079	0.641 (0.487-0.795)				
	wavelet_LLH_firstorder_Skewness.ZQ	0.086	0.638 (0.476-0.799)				
combined model	original_firstorder_Kurtosis.ZQ	0.001*	0.751 (0.613-0.899)				
	wavelet_HHL_glcm_Imc2.T2	0.002*	0.747 (0.601-0.884)				
	log_sigma_2_0_mm_3D_gldm_DependenceNonUniformityNormalized.ZQ	0.004*	0.727 (0.584-0.869)				
	wavelet_LHL_firstorder_Mean.ZQ	0.005*	0.725 (0.583-0.868)				
	wavelet_HHL_firstorder_Mean.T2	0.005*	0.721 (0.579-0.863)				
	original_glrlm_LongRunHighGrayLevelEmphasis.ZQ	0.014*	0.702 (0.556-0.849)				
	wavelet_HLL_glszm_ZoneEntropy.ADC	0.015*	0.695 (0.548-0.842)				
	wavelet_HHL_glcm_MCC.ADC	0.017*	0.691 (0.536-0.845)				
	wavelet_LHH_glcm_MCC.ADC	0.020*	0.687 (0.530-0.843)				
	original_shape_Elongation.ZQ	0.038*	0.667 (0.519-0.814)				

*p<0.05

**Table 6:** Diagnostic performance of four different models

model	Accuracy (95% confidence interval)	Sensitivity	Specificity	PPV	NPV
T2WI model	0.637 (0.501 - 0.760)	0.625	0.653	0.689	0.586
ADC model	0.741 (0.609 - 0.847)	0.733	0.75	0.759	0.724
CE_T1WI model	0.741 (0.609 - 0.847)	0.769	0.718	0.689	0.793
combined model	0.759(0.628 - 0.861)	0.778	0.741	0.724	0.793

Abbreviations: PPV positive predictive value, NPV negative predictive value

## Abbreviations

O-RADS: Ovarian-adnexal reporting and data system
MRI: Magnetic Resonance Imaging
T1WI: T1 Weighted image
T2WI: T2 Weighted image
DWI: Diffusion Weighted Imaging
ADC: Apparent Diffusion Coefficient
CE: Contrast-enhanced
ROI: Region of Interest
ROC: Receiver Operating characteristic Curve
AUC: Area Under the Receiver Operating characteristic Curve
ICC: The intra-class correlation coefficient
mRMR: minimum Redundancy Maximum Relevance
RF: Random forest
TA: Texture analysis
CI: Confidence interval
SD: Standard deviation
PPV: Positive predictive value
NPV: Negative predictive value
GLCM: Gray Level Co-occurrence Matrix
GLRLM: Gray Level Run Length Matrices
GLSZM: Gray Level Size Zone Matrix
GLDM: Gray Level Dependence Matrix
CA125: Carbohydrate antigene125
AFP: Alpha fetoprotein
CEA: Carcinoembryonic antigen
